# *PDE4B* gene polymorphism in Russian patients with panic disorder

**DOI:** 10.3934/genet.2019.3.55

**Published:** 2019-08-20

**Authors:** Alena V Malakhova, Olga I Rudko, Vladimir V Sobolev, Artemii V Tretiakov, Elena A Naumova, Zarema G Kokaeva, Julia E Azimova, Eugene A Klimov

**Affiliations:** 1 Lomonosov Moscow State University, Faculty of Biology, Moscow, Russia; 2 I.I. Mechnikov Research Institute for Vaccines and Sera, Laboratory of molecular immunology, Moscow, Russia; 3 University Diagnostic Laboratory LLC, Moscow, Russia; 4 Centre of Theoretical Problems of Physico-Chemical Pharmacology, Laboratory of Physicochemical and Genetic Problems of Dermatology, Russian Academy of Sciences, Moscow, Russia; 5 University headache clinic LLC, Moscow, Russia; 6 The Institute of General Pathology and Pathophysiology, Laboratory of Fundamental and Applied Pain Problems, Moscow, Russia; 7 Center of Experimental Embryology and Reproductive Biotechnologies, Russian Academy of Sciences, Moscow, Russia

**Keywords:** *PDE4B* gene, rs10454453, rs502958, rs1040716, panic disorder, genetic association

## Abstract

**Background:**

Panic disorder is a complex disease of unclear etiology but with an apparent genetic component. *PDE4B* gene product is involved in many cell processes owing to its function-regulation of the level of a second messenger cAMP. *PDE4B* gene polymorphism has been shown to be associated with some mental disorders including panic disorder.

**Aims:**

The goal of our study was to evaluate the role of 3 SNPs in the *PDE4B* gene in the development of panic disorder.

**Methods:**

94 patients diagnosed with panic disorder according to the DSM-IV criteria were enrolled in the study. The population control group included 192 subjects. Genotyping was carried out by real-time PCR with TaqMan probes.

**Results:**

The investigated substitutions are not associated with panic disorder in general and in female/male cohorts (p > 0.05). The analysis of complex genotypes demonstrated two protective complex genotypes (rs1040716:A, T + rs10454453:A + rs502958:A and rs1040716:A, T + rs502958:A) associated with panic disorder in general regardless of the patient's gender (p < 0.05). These genotypes did not correlate with the patient's sex.

**Conclusions:**

We found two complex protective genotypes associated with panic disorder. This can be due to the fact that predisposition to the disease are associated with other genes, while *PDE4B* gene polymorphism reduces their effect.

## 1. Introduction

### 1.1. Panic disorder

Panic disorder (PD) is a disorder characterized by sudden and recurrent attacks of anxiety, fear of death, fear of becoming insane accompanied by physical symptoms (dizziness, excessive sweating, palpitations and chest pain, shivering, feeling of shortness of breath, nausea and abdominal pain, paresthesia and derealization). Despite short duration (several minutes) of attacks, panic disorder has a significant impact on the quality of life of patients. As the diseases progresses, anxiety and fear of having a panic attack start persisting and limiting the patients' daily activities. Frequent panic attacks (more than 1 attack per week) cause the most significant impact on the patient's adjustment.

Evidence of direct inheritance of the disorder from generation to generation has been obtained (the disorder is observed in 15–17% of relatives of patients with PD; 85–90% concordance in monochorionic twins; heritability 48%) [1]. However, molecular genetic basis of this polygenic disorder has not been well studied [2].

### 1.2. Phosphodiesterases

Phosphodiesterases (PDE) regulate intracellular concentration of cyclic adenosine monophosphate (cAMP), second messenger, which plays a role in intracellular signaling and is involved in the processes of learning, memory and mood swings [3],[4]. PDE blockade in the cell has been shown to result in increased cAMP concentration, which affects many cellular processes. PDE4 subtype B (PDE4B) is coded by a gene located in the area of 1p31.2 that includes 17 exons. Changes in the *PDE4B* gene expression contributes to the change in intracellular cAMP concentrations, which correlates with various psychiatric disorders. There was a report regarding a patient with schizophrenia and his relative with a chronic psychiatric disorder who were found to have a balanced translocation impairing the *PDE4B* gene structure [5]. Correlation between *PDE4B* gene polymorphism and schizophrenia was demonstrated [6]–[9], and so was the correlation with bipolar disorder [9] and depression [10]. The expression of *PDE4B* isoforms assessed post mortem in the brain of patients with schizophrenia and bipolar disorder differs from that in the control group [7].

### 1.3. PDE4B involvement in mental disorders

PDE4B has also been shown to interact with DISC1, a protein that plays an important role in synaptic plasticity and is a potential susceptibility factor for mental disorders, including schizophrenia, schizoaffective disorder, bipolar disorder, depression and anxiety [11]. DISC1 has been shown to regulate the activity of PDE4B in mitochondria and the cytosol of neurons, while N-terminal site-directed mutations in the *DISC1* gene lead to deterioration of synaptic plasticity [12].

There is evidence of PDE4B involvement in the regulation of affective disorders, anxiety and depression. PDE4B expression has been found in the amygdaloid body, pituitary gland and the anterior cortex [13], which are key regions responsible to anxiety-related behavior and response to stress [14]. cAMP signaling has been also shown to regulate anxiety-related behavior [15]. In this process, PDE4B might play a role of a critical signaling checkpoint [16]. PDE4B inhibitor rolipram produces an effect on behavior that is similar to that of antidepressants [3] and anxiolytics [16],[17]. Other selective PDE4B inhibitor GSK356278 also demonstrates a pronounced anxiolytic effect [18]. Long-term treatment with anxiolytics reduces the PDE4B expression and increases neurogenesis in mice [19],[20]. Benzodiazepine anxiolytic diazepam also inhibits the PDE4B expression [21].

However, on the other side, artificial decrease in the level of *Pde4b* gene expression in knockout mice (-/-) is accompanied by the decreased PDE activity in the brain regions causing the increase in plasma corticosterone levels and producing anxiogenic effects in behavioral testing [22].

Despite a large number of assumptions, the only associative study concerning the connection between the polymorphic sites of *PDE4B* gene and PD pathogenesis was conducted in 2010. A statistically significant association of rs10454453 substitution with the development of panic disorder was shown in a Japanese population [23].

To evaluate the role of *PDE4B* polymorphism in panic disorder development we analyzed not only the substitutions rs10454453 (NM_002600.3:c.282-40611C>A, intron 6) and rs502958 (NM_002600.3:c.585-539A>T, intron 10) but also rs1040716 (NM_002600.3:c.635-20601A>T, intron 11), for which a correlation with schizophrenia was demonstrated [24]. We evaluated the role of 3 SNPs in the *PDE4B* gene in the development of panic disorder and found two protective complex genotypes associated with panic disorder.

## 2. Subjects and methods

### 2.1. Patients with panic disorder

The study included patients with the panic disorder diagnosis under DSM-IV criteria. The study included only those patients that were subject to frequent panic attacks (at least one per week). The sample size was 94 patients with panic disorder according to criteria ICD-10 (F 41.0)—75 female and 19 male. All the patients live in Moscow and Moscow region and were referred to University headache clinic for treatment. Patient recruitment was carried out from 2010 to 2017. The patient was included in the study for at least after six months of permanent observation. Diagnosis was confirmed by a psychiatrist. All the patients gave their informed consent to the participation in the study. The study is approved by the Local ethical committee of Vavilov Institute of General Genetics of Russian Academy of Sciences. DNA samples extracted from the whole blood of unscreened volunteers residing in Moscow and Moscow region were used as the population control (n = 192, 108-female, 84-male).

### 2.2. DNA extraction and PCR

Samples of DNA were extracted from whole blood of patients. DNA was extracted according to protocol to commercial DNA Magna™ DNA Prep 200 kit (Isogen Laboratory LLC, Moscow, Russia). Genotypes were identified by real-time PCR method. The PCR was conducted according to protocol of commercial kit qPCRmix-HS (Evrogen JSC, Moscow, Russia). Primers were synthesized by DNA-Synthesis LLC (Moscow, Russia). Primers and fluorescent probes sequences are listed in Table 1.

PCR testing was performed under similar conditions for all substitutions: Preliminary denaturation 94 °C-1 min, 30 cycles (94 °C-30 sec, 63 °C-1 min, 72 °C-1 min).

**Table 1.** The sequences of primers and allele-specific fluorescent probes to substitutions in the *PDE4B* gene used in the study.

rs1040716	F: CGTAAGGAAGGAGAAGCTCTGTATG
	R: GTGCCCTAATGCCAGTGGAAGA
	T: FAM_CAGAGCAGATCCCTATATGC_BHQ1
	A: VIC_CAGAGCAGAACCCTATATGC_BHQ1
rs502958	F: AAGGTCACACAACCACTGGGAAC
	R: CAGTACTATGGGAACATGGGTTTGC
	T: FAM_ GAACAGAATTTTCATGGAGGAAC_BHQ1
	A: VIC_GAACAGAATTTACATGGAGGAAC_BHQ1
rs10454453	F: AGAGATTGCATGGTCCACTAGCTCAG
	R: CCATGATAAGCTGGGCTGTAATGCA
	C: FAM_GTCTTTGAATCCCTAGCATGTA_BHQ1
	A: VIC_TTGTCTTTGAATCACTAGCATGTAA_BHQ1

### 2.3. Data analyses

Statistical data analysis was performed using WinPepi software, module COMPARE2 (comparison of two independent groups) [25]. The presence of the association was determined by the p-value of two-tailed Fisher test (p < 0.05). APSampler software v3.6.1 was used for the search of polygenic associations (complex genotypes) [26]. The program algorithm identifies all the phenotype-associated alleles/genotypes combinations, data with a permutation (Westfall-Young) p-value < 0.05 were considered reliable.

## 3. Results and discussions

The obtained frequencies of genotypes and alleles of the studied substitutions in the study group and the control group, as well as in the groups divided by gender are presented in Tables 2 and 3.

All substitutions were tested for compliance with the Hardy-Weinberg equilibrium (p < 0.05) in the studied samples. No associations of the PD development and genotypes or alleles of the studied substitutions were found (two tailed Fisher's test p > 0.05) for the general group of patients and the subgroups (male and female).

We used APSampler 3.6.1 software to search for combinations of genotypes and/or alleles associated with PD. In general group, two combinations of genotypes that complied with the permutation test were revealed (Table 4). No genotype combinations for male or female groups were revealed.

**Table 2.** The frequencies of genotypes of the studied substitutions in *PDE4B* gene in the group of patients with panic disorder (PD) and the control group (Cont) as well as the subgroups.

SNP and genotypes		All samples		Female		Male	
		Cont	PD	Cont	PD	Cont	PD
rs502958	AA	0.289	0.261	0.148	0.240	0.167	0.316
	AT	0.505	0.500	0.574	0.427	0.607	0.316
	TT	0.205	0.239	0.278	0.333	0.226	0.368
rs1040716	AA	0.156	0.255	0.352	0.400	0.386	0.316
	AT	0.589	0.404	0.537	0.467	0.506	0.526
	TT	0.255	0.340	0.111	0.133	0.108	0.158
rs10454453	AA	0.366	0.383	0.271	0.247	0.313	0.316
	AC	0.524	0.479	0.551	0.493	0.446	0.526
	CC	0.110	0.138	0.178	0.260	0.241	0.158
		n = 192	n = 94	n = 108	n = 75	n = 84	n = 19

**Table 3.** The frequencies of alleles of the studied substitutions in *PDE4B* gene in the group of patients with panic disorder (PD) and the control group (Cont) and the subgroups.

SNP and alleles		All samples		Female		Male	
		Cont	PD	Cont	PD	Cont	PD
rs502958	A	0.471	0.452	0.379	0.433	0.386	0.443
	T	0.529	0.548	0.621	0.567	0.614	0.557
rs1040716	A	0.386	0.443	0.511	0.543	0.521	0.532
	T	0.614	0.557	0.489	0.457	0.479	0.468
rs10454453	A	0.521	0.532	0.461	0.442	0.471	0.452
	C	0.479	0.468	0.539	0.558	0.529	0.548

There were two statically significant genotype combinations that were protective in general group of patients with PD (OR < 1). The heterozygous genotype AT of rs1040716 substitution and the allele A of rs502958, as well as the A allele of substitution rs10454453 were found to play a major role. Interestingly, the A allele of rs1040716 is recessively inherited and is link, however, there is no significant evidence of its correlation with PD (two tailed Fisher's test p = 0.054).

To assess the functional role of SNPs using *in silico* analysis by Human Splicing Finder 3.1 software (http://www.umd.be/HSF3/) [27]. For rs502958 A>T substitution, a new donor splicing site (+53.54%) was found in the presence of T allele that could possibly extend the exon 9 by 531 nucleotides. The new site appears to be specific for the SRSF6 splicing factor (value 74.56). For rs1040716 A>T substitution, a new splicing site in presence of T allele was found (+68.30%); the site appears to be specific for the SRSF2 splicing factor (value 79.72). rs10454453 C>A substitution had no effect on the splicing. Thus, both substitutions in the complex genotypes may affect the *PDE4B* mRNA splicing leading to the formation of non-functional products.

**Table 4.** The result of search for associations of combinations of genotypes and/or alleles with PD.

Genotypes	Fi (p)	OR	CI(95%)	P1000 (p)
rs1040716:A,T; rs10454453:A; rs502958:A	5.67E-4	0.393	0.225–0.687	0.020
rs1040716:A,T; rs502958:A	6.73E-4	0.411	0.241–0.702	0.027

Note: Fi: Fisher's exact p-value; OR: odds ratio; CI (95%): confidence intervals p < 0.05; P1000 (p): permutation (Westfall-Young) p-value (1000 permutation).

Impaired PDE4B functioning causes the excess of cAMP and hyperactivation of cAMP-dependent signaling pathways. However, it remains unclear the signaling pathway of which receptor will be changed in altered protective complex genotype carriers who were diagnosed with PD.

The only study that evaluated the association of SNPs in the *PDE4B* gene with panic disorder demonstrated the relationship between a rs10454453 substitution and PD development. Significant correlation was observed in the female subpopulation only [23]. In a Japanese population of PD patients, this substitution was shown to be associated with PD both separately and in combination with rs10454453, rs6588190, rs502958 and rs1040716 (associated haplotype C-T-T-A, respectively). Our study identified complex protective genotypes, with rs1040716 and rs502958 substitutions contributing the most (complex AT-A genotype, respectively). The rs10454453 substitution adds a protective effect to the combination of all three substitutions (OR = 0.393, permutation p-value = 0.020). A comparison of the results obtained by Otowa et al. and our findings showed that complex protective genotypes (AT-A-A) identified in our study were opposite to those identified in the Japanese study (A-C-T). This suggests the role of *PDE4B* polymorphism in PD pathogenesis. Moreover, there seems to be a change in splicing regulation since PD-related SNPs can change the splicing efficiency. Patients with schizophrenia have been shown to have a decreased number of PDE4B4 and PDE4B2 isoforms, while patients with bipolar disorder have been shown to have a decreased number of PDE4B3 in the cerebellum [7]. The substitutions studied by us and Otowa et al. are located in the splicing regulatory element of one of *PDE4B* isoforms, which may affect the expression efficiency of this isoform (PDE4B2). The mirror data obtained in these two studies (our study and [23]) may be explained by sample differences in respect of both ethnicity and clinical data of the patients. However, our study confirms and complements the results obtained by Otowa et al.

However, *PDE4B* polymorphism is associated with alcohol consumption [28]. A relationship between alcohol consumption and panic disorder in humans [29]–[31] has been shown.

The rs1040716 substitution, in addition to being associated with PD [23], has also been shown to be associated with schizophrenia [24],[32],[33], however, other studies did not find any associations between rs1040716 and schizophrenia [8],[34]. SNPs rs10454453 and rs502958 have been studied in relation to panic disorder only [23].

We were not able to find any genotypes that predispose to the development of panic disorder. This can be due to the fact that predisposition to the disease are associated with other genes, while *PDE4B* gene polymorphism reduces their effect. Further research is needed to determine the functional role of these substitutions in the PD development.

## 4. Conclusions

We estimated the frequencies of three SNPs in the *PDE4B* gene in a group of patients with panic disorder and a control group. Two complex protective genotypes (OR < 1) associated with panic disorder were revealed: rs1040716:A,T + rs10454453:A + rs502958:A (p = 0.020) and rs1040716:A, T + rs502958:A (p = 0.027). These genotypes did not correlate with the patient's sex.
